# Developing a High-Throughput SNP-Based Marker System to Facilitate the Introgression of Traits From *Aegilops* Species Into Bread Wheat (*Triticum aestivum*)

**DOI:** 10.3389/fpls.2018.01993

**Published:** 2019-01-24

**Authors:** Alexandra M. Przewieslik-Allen, Amanda J. Burridge, Paul A. Wilkinson, Mark O. Winfield, Daniel S. Shaw, Lorna McAusland, Julie King, Ian P. King, Keith J. Edwards, Gary L. A. Barker

**Affiliations:** ^1^Life Sciences, University of Bristol, Bristol, United Kingdom; ^2^Plant Sciences, Sutton Bonington Campus, Leicestershire, United Kingdom

**Keywords:** *Aegilops*, wheat, genotyping array, single nucleotide polymorphism (SNP), introgression, wheat relative

## Abstract

The genus *Aegilops* contains a diverse collection of wild species exhibiting variation in geographical distribution, ecological adaptation, ploidy and genome organization. *Aegilops* is the most closely related genus to *Triticum* which includes cultivated wheat, a globally important crop that has a limited gene pool for modern breeding. *Aegilops* species are a potential future resource for wheat breeding for traits, such as adaptation to different ecological conditions and pest and disease resistance. This study describes the development and application of the first high-throughput genotyping platform specifically designed for screening wheat relative species. The platform was used to screen multiple accessions representing all species in the genus *Aegilops*. Firstly, the data was demonstrated to be useful for screening diversity and examining relationships within and between *Aegilops* species. Secondly, markers able to characterize and track introgressions from *Aegilops* species in hexaploid wheat were identified and validated using two different approaches.

## Introduction

**Figure 1:**
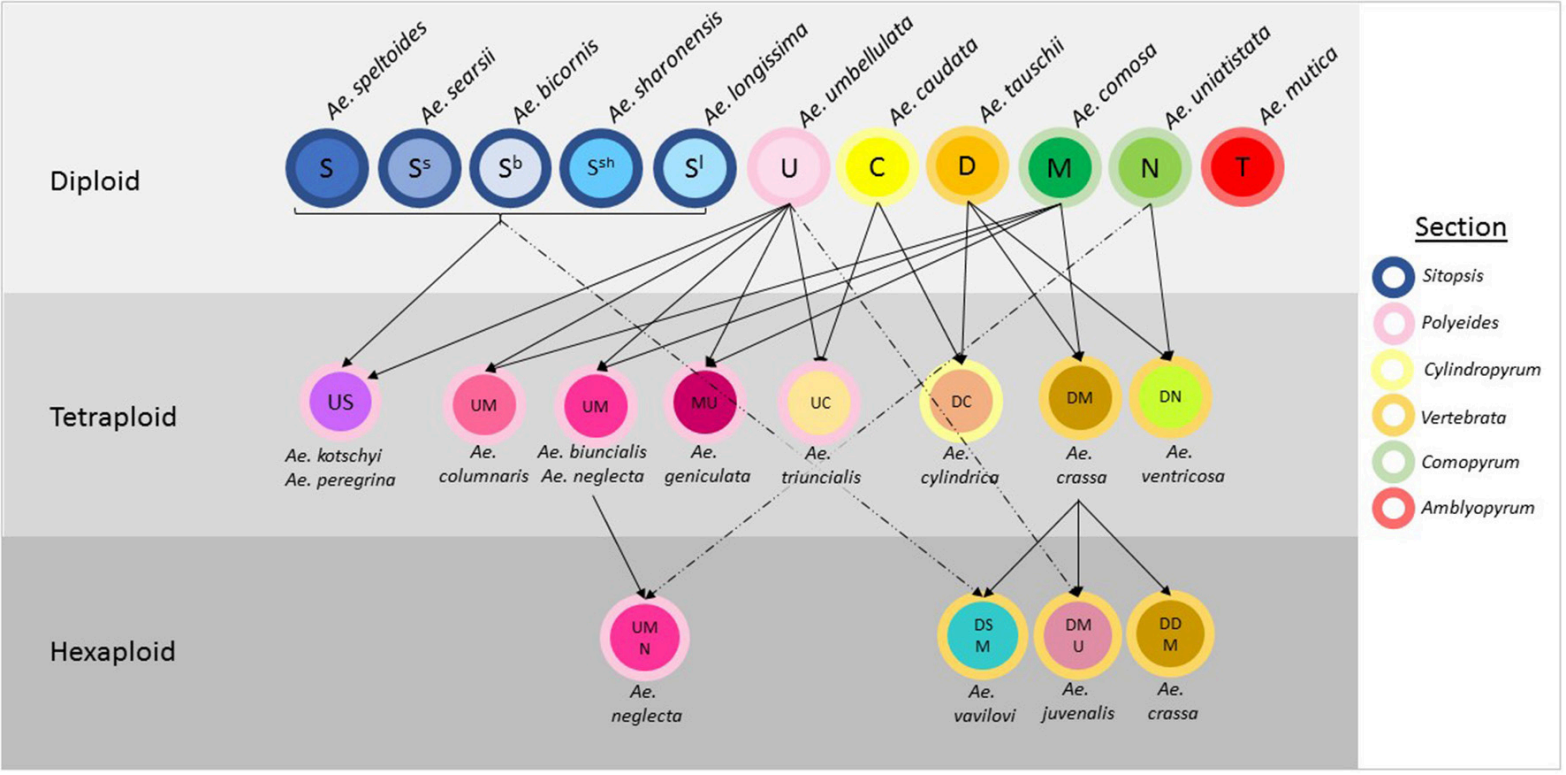
Relationships between species in the genus Aegilops. The genome classification of each species is indicated within circles representing the species and arrows designate hybridisations between species. The color of the outline of each circle represents the section the species is allocated to.

*Aegilops* is a genus of Eurasian annual grasses in the Poaceae known as the goatgrasses. There are 23 species within *Aegilops*; these species represent six different genome types (D, S, U, C, N, and M) and three different ploidy levels (diploid, tetraploid, and hexaploid) (The Plant List, 2013; Molnár et al., 2016; Figure 1). The genus *Aegilops* is the most closely related to the genus *Triticum*, which contains *Triticum aestivum* (bread wheat) and other domesticated wheats. Researchers have suggested that *Aegilops* and *Triticum* should be combined into a single evolutionary complex or even the same genus (Yamane and Kawahara, 2005). The close genetic relationship is evidenced by the numerous hybridisations that occur between members of both genera and by the presence of *Aegilops* in the evolutionary history of many *Triticum* species. Where geographic distributions are similar, gene flow has occurred between species; some species, such as *Aegilops cylindrica* have spread with wheat and have become uncontrollable weeds in wheat (Donal and Ogg, 1991). If treated separately, *Aegilops* appears to be basal to *Triticum*, with evidence indicating the genus' *Triticum* and *Aegilops* diverged an estimated 4 million years ago (Huang et al., 2002).

*Aegilops* has been divided into six sections based on morphological and genetic analysis. These are *Sitopsis* (Jaubert and Spach, 1850–1853) Zhuk., *Amblyopyrum* (Jaubert and Spach, 1850–1853) Eig, *Polyeides* Zhuk., *Cylindropyum* (Jaubert and Spach, 1850–1853) Zhuk., *Comopyrum* (Jaubert and Spach, 1850–1853) Zhuk., *Vertebrata* Zhuk (Table 1; Zhukovsky, 1928; Eig, 1929; Yamane and Kawahara, 2005; Schneider et al., 2008; Wang et al., 2013). *Aegilops mutica (syn. Amblyopyrum muticum)* has been separated by some researchers and placed into a monospecific genus called *Amblyopyrum* (Van Slageren, 1994) but for the purposes of this study has been included with other members of *Aegilops* for analysis. *Ae. speltoides* is thought to be the closest relative to the wheat B-genome and is also the donor of the G-genome of *Triticum timopheevii* (Dvorak et al., 1998a; Feldman, 2001)*. Ae. tauschii* is the progenitor of the wheat D-genome, hybridizing with the AB-genome progenitor ~10,000 years ago to produce hexaploid bread wheat (McFadden and Sears, 1946). This rare hybridization event is thought to have only occurred once or a small number of times resulting in a severe genetic bottleneck (Charmet, 2011). Further inbreeding and domestication pressures have resulted in a narrow gene pool for modern bread wheat breeding.

**Table 1:** Details of germplasm used in study.

**Species**	**Section**	**Genome**	**Ploidy**	**Number of accessions**	**Number of SNPs within species**	**Number of SNPs compared to *Triticum aestivum***
*Aegilops mutica*	*Amblyopyrum*	TT	2	12	15,617	33,816
*Aegilops comosa*	*Comopyrum*	MM	2	14	16,103	33,305
*Aegilops uniaristata*	*Comopyrum*	NN	2	6	9,190	33,120
*Aegilops caudata*	*Cylindropyrum*	CC	2	7	11,668	33,433
*Aegilops cylindrica*	*Cylindropyrum*	DDCC	4	7	13,147	30,052
*Aegilops biuncialis*	*Polyeides*	UUMM	4	8	13,040	33,129
*Aegilops columnaris*	*Polyeides*	UUMM	4	7	13,953	33,419
*Aegilops geniculata*	*Polyeides*	MMUU	4	5	12,412	33,772
*Aegilops kotschyii*	*Polyeides*	SSUU	4	8	11,658	33,024
*Aegilops neglecta*	*Polyeides*	UUMM/UUMMNN	4 or 6	6	17,373	33,306
*Aegilops peregrina*	*Polyeides*	UUSS	4	9	16,428	33,127
*Aegilops triuncialis*	*Polyeides*	UUCC	4	17	17,265	33,378
*Aegilops umbellulata*	*Polyeides*	UU	2	15	15,254	33,365
*Aegilops bicornis*	*Sitopsis*	SS	2	5	17,817	34,085
*Aegilops longissima*	*Sitopsis*	SS	2	10	15,835	33,093
*Aegilops searsii*	*Sitopsis*	SS	2	13	10,575	32,804
*Aegilops sharonensis*	*Sitopsis*	SS	2	13	18,220	33,339
*Aegilops speltoides*	*Sitopsis*	SS	2	38	24,524	32,401
*Aegilops crassa*	*Vertebrata*	DDMM/DDMMMM	4 or 6	7	11,798	30,103
*Aegilops juvenalis*	*Vertebrata*	DDMMUU	6	5	12,990	31,120
*Aegilops tauschii*	*Vertebrata*	DD	2	22	21,867	31,212
*Aegilops vavilovii*	*Vertebrata*	DDSSMM	6	5	13,893	30,969
*Aegilops ventricosa*	*Vertebrata*	DDNN	4	11	14,631	30,211

The genus *Aegilops* promises to be an important resource for wheat breeding as it harbors a high level of genetic diversity, particularly with relation to adaptation to different ecological conditions and pest and disease resistance. All *Aegilops* species are undomesticated and have wide geographic distributions and natural variation (Ostrowski et al., 2016). *Aegilops* contains species belonging to the secondary gene pool of wheat, meaning they have a genome homologous with wheat and conventional crossing may be used to transfer genes to wheat (*Ae. tauschii* and *Ae. speltoides*). Other more distantly related members of the genus belong to the tertiary gene pool of wheat and may need specific breeding techniques for gene transfers to wheat, although crosses between the two genera have been reported to occur naturally (Popova, 1923; Leighty and Taylor, 1927; Schneider et al., 2008). Interspecific hybridization between bread wheat and members of the genus *Aegilops* has been used historically in wheat breeding to confer beneficial traits from *Aegilops* into bread wheat. These include resistance to rusts, powdery mildew, eyespot, nematodes, hessian fly and wheat aphid (see Schneider et al., 2008 for a full review). The genus *Aegilops* is a potential source of further genes conferring agronomically valuable traits, such as drought tolerance, salt tolerance, heat tolerance, tolerance to toxicity and nutritional and bread-making quality traits of potential use in plant breeding (Molnár et al., 2004; Colmer et al., 2006; Schneider et al., 2008; Kilian et al., 2011).

Advances in genome sequencing over the last decade have had huge impacts on our knowledge of the large and complex hexaploid wheat genome and our ability to develop molecular markers (Uauy, 2017; The International Wheat Genome Sequencing Consortium (IWGSC), 2018). The knock-on effects of these developments have been seen in the breeding lines developed, the widespread adoption of molecular markers in breeding programmes and the development of new breeding techniques, such as genomic selection (Schneider et al., 2008; Bassi et al., 2016). At the same time there has been recognition of the importance of pre-breeding programmes specifically targeted at introducing genetic diversity from exotic sources, such as landraces and wheat relatives (Moore, 2015). The introduction of such diverse material has necessitated the development of specific molecular markers that are able to identify and characterize wheat relative DNA in the wheat genome (Winfield et al., 2016).

With the development of genomic tools and technologies enabling precise and efficient breeding *Aegilops* promises to be an increasingly important resource of genetic diversity in future wheat breeding. A potential drawback of utilizing wide crosses to introduce diversity in this way is the inclusion of large non-recombining blocks from a relative into the wheat genome. However, with the development of genomic technologies, improved crossing techniques and gene editing technologies it is becoming possible to target genomic regions with increased precision. To enable these techniques to be employed successfully there is a requirement for increasingly dense and precise molecular markers, which can be utilized in a high-throughput manner. A key challenge is to develop markers to track introgressed DNA in the wheat genetic background. This study describes the identification, validation and use of markers systems for facilitating the introgression of *Aegilops* species into hexaploid wheat. The wide range of species used in the study represent the three different ploidy levels and six genome types found within the genus. The markers developed have enabled the detection of *Aegilops* introgressions in newly developed lines with examples of how these markers have been deployed in different introgression projects.

## Materials and Methods

### Germplasm

The accessions grown for DNA extraction (listed in Supplementary File 1) were grown in peat-based soil in pots and maintained in a glasshouse at 15–25°C with 14-h light, 8-h dark. Leaf tissue was harvested 4 weeks after germination, frozen in liquid nitrogen and stored at −20°C prior to nucleic acid extraction. Genomic DNA was prepared using a phenol–chloroform extraction method (Burridge et al., 2017), treated with RNase-A (QIAGEN Ltd., Manchester, UK) according to the manufacturer's instructions and purified using the QiaQuick PCR purification kit (QIAGEN Ltd).

### Genotyping

The original SNP collection consisted of 819,571 SNPs obtained from genic sequences derived via targeted capture re-sequencing of numerous wheat lines and validated on the Axiom® HD Wheat Genotyping Array (Winfield et al., 2016; Affymetrix UK Ltd, High Wycombe, UK; EVA accession PRJEB29561). The most informative 36,711 SNPs were selected for inclusion on The Axiom® Wheat-Relative Genotyping Array, based on data from screening ten wild relative species (*Ae. mutica, Ae. speltoides, Aegilops. tauschii, Triticum timopheevii, T. urartu, Secale cereale, Thinopyrum bessarabicum, Th. elongatum, Th. Intermedium*, and *Th. Ponticum*, Supplementary File 2; http://www.cerealsdb.uk.net/cerealgenomics/CerealsDB/axiom_download.php). Markers were selected to maximize polymorphism between the relative species' and wheat. Markers chosen behaved in a co-dominant manner making them potentially useful for identifying heterozygous calls in wheat-relative crosses (Allen et al., 2012). The Axiom® Wheat-Relative Genotyping Array was used to screen 278 *Aegilops* accessions using the Affymetrix GeneTitan™ system according to the procedure described by Affymetrix (Axiom®2.0 Assay Manual Workflow User Guide Rev3). Allele calling was carried out using the Affymetrix proprietary software packages Axiom Analysis Suite™. A variant Dish QC threshold of 0.75 was used instead of the default value (0.8) to account for the lower call rates typically obtained from hybridizing wheat relatives and progenitors to the array (Winfield et al., 2016).

The probes on the array are biallelic; for each locus there is a maximum of three calls possible (AA, AB, BB or 0, 1, 2) possible. The clustering pattern for each locus will depend upon the other lines that have been screened. For diploid species the clustering and genotype calling is straightforward, however screening polyploid lines is more complex. Although the intended application of the array is for specific intraspecific crosses where discrete populations are analyzed separately it is also possible to use the array to screen more diverse collections. For more complex populations or collections, a recommended approach would be to focus on particular loci of interest that produce clear clustering patterns. If necessary, it would be possible to examine the behavior of this subset of probes on the array and relate these to known genotypes. An important factor to note is whether the probe is “co-dominant” and interacts with just the genome of interest or if homeologous genomes also hybridize which will complicate the clustering pattern (Allen et al., 2012). Generally, probes that preferentially or specifically hybridize to a single genome will give higher quality clustering patterns even when screening diverse lines and users could preferentially choose these for further analysis.

For example, the accessions of DDMMUU genome have the genotype ABAAAA and BBAAAA and an accession of the DD genome has the genotype AB. For a dominant probe you would get the following calling pattern: 0 = ABAAAA, 1 = BBAAAA, 2 = AB (assuming the interaction of the MM and UU genomes also). For a co-dominant, D-genome specific probe you would get 0 = BB, 1 = AB. Introducing more different lines with different genome compositions could further complicate the clustering pattern.

Assignment of a physical map position to the SNP markers was achieved by BLAST searching the probe sequences to the International Wheat Genome Sequencing Consortium (IWGSC) whole genome assembly v1.0. For further analysis (see below) data were screened for quality using the monomorphic and call rate filtering of Axiom Analysis Suite. High quality probes taken forward for PCoA and phylogenetic analysis had a call rate of 80% or higher.

### Dimensionality Reduction

A distance matrix was generated from the genotype scores using R package SNPRelate (Zheng et al., 2012). The proportion of variance for the first six eigenvalues was as follows: 26.45, 15.93, 7.21, 4.60, 4.42, 3.97. The first two eigenvalues with over 42% of the variance were plotted as a PCA plot.

### Phylogenetic Analysis

Evolutionary relationships between Aegilops varieties were investigated using the genotype calls from the wheat relative genotyping array. The SNPhylo pipeline (version 20160204; Lee et al., 2014) was used to construct phylogenetic trees based on a haplotype map of 278 Aegilops varieties that was derived from the genotype calls. The SNPhylo pipeline removed 13,253 lines of low quality data from the hapmap file, using the software's default parameters. Low quality data was defined as monomorphic, a MAF score of <0.1 or where 10% or more of the varieties had missing data. *Aegilops mutica* was assigned as an outgroup to root the tree based upon PCA results and putative reclassification to a separate genus from *Aegilops*. The hapmap file was then submitted to the SNPhylo pipeline, and a maximum likelihood tree was generated with a bootstrapping value set to 10,000. The Newick strings generated by SNPhylo were imported into the R package ggtree (version 1.2.17; Yu et al., 2017) which was used to construct a circular dendrogram.

### Introgression Detection

The identification of putative introgressions was performed by comparing the genotype calls of hexaploid lines to Aegilops accessions over a 10 SNP window and calculating a percentage match. Analysis of control introgression lines indicated that a match of 40% or higher within the 10 SNP window was indicative of an introgression in the wheat background. This threshold was chosen based on the screening of known introgressions, such as 1B/1RS.

## Results

### Diversity Within the Genus *Aegilops*

Of the 36,711 SNPs on the wheat relative array 34,602 (94.3%) were polymorphic in the entire collection of *Aegilops* accessions used in the study (Supplementary File 2). The SNPs fell into the following classification categories: Poly High Resolution, 18.8%; No Minor Homozygote, 10.3%; Off Target Variant, 19.3%; Mono High Resolution, 5.6%; Call Rate Below Threshold, 5.6%; Other, 40.3%. The number polymorphic within each species ranged from 9,190 (25.0%; *Ae. uniaristata*) to 24,524 (66.9%; *Ae. speltoides*) and was related to the number of accessions genotyped (*R*^2^ = 0.56) (Table 1). This was explored further by selecting four random subsets of five accessions for each of *Ae. speltoides* and *Ae. tauschii*. The number of polymorphisms detected in the *Ae. speltoides* subsets ranged from 10,842 to 12,366 and for *Ae. tauschii* ranged from 6,555 to 11,866. These figures are comparable to the other species sampled and suggest that the number of accessions has a clear effect on the number of polymorphisms detected rather than the species in question. The number of SNPs polymorphic between each species and the wheat samples genotyped ranged from 30,052 (81.9%; *Ae. cylindrica*) to 34,085 (92.8%; *Ae. bicornis*) and there appeared to be no relationship (*R*^2^ = 0) between the number of accessions genotyped and the number of polymorphisms detected when compared with wheat. There was no significant relationship between the number of polymorphic SNPs within a species compared to between the species and wheat (*R*^2^ = 0.0047).

**Figure 2:**
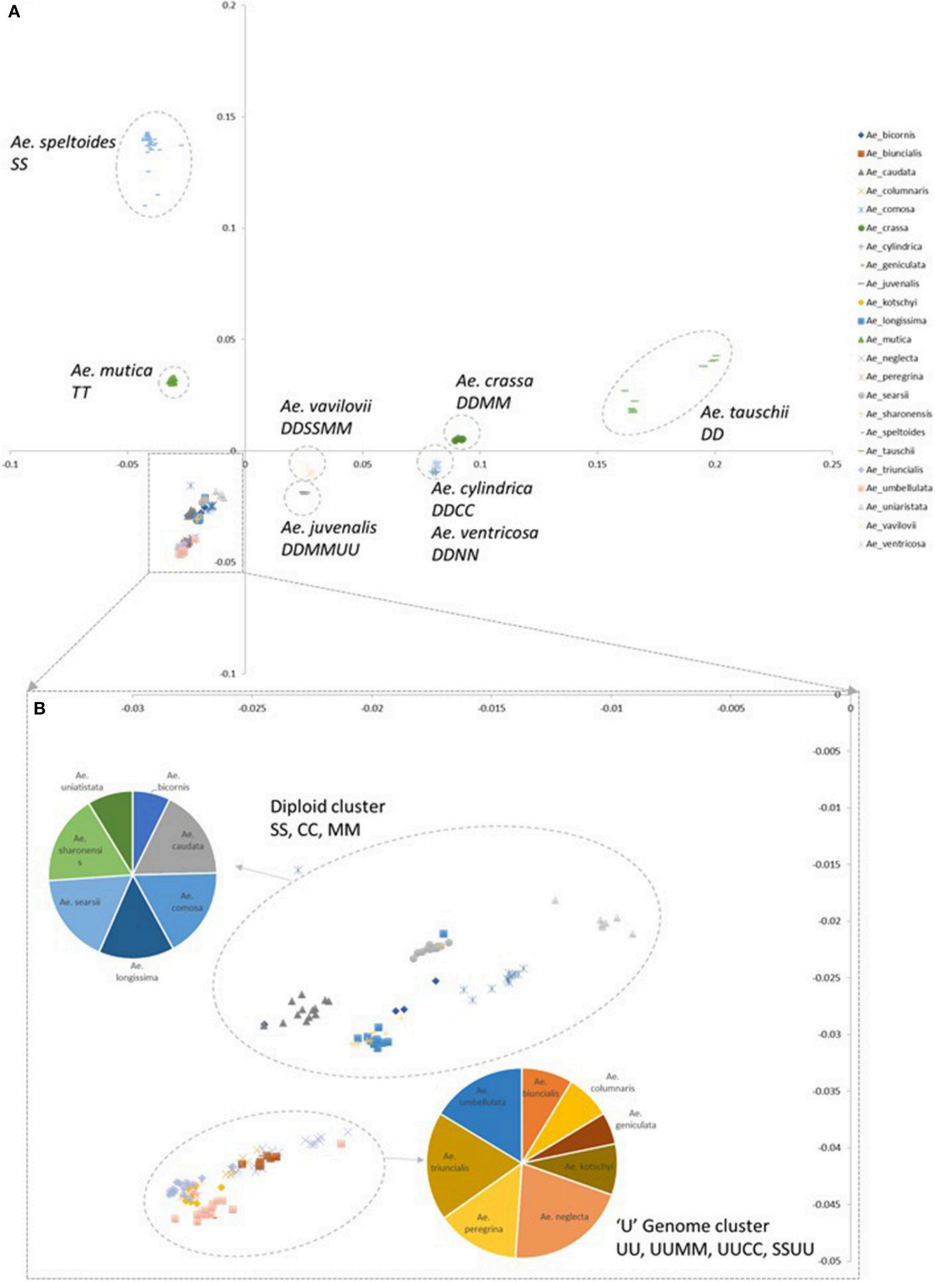
Principal coordinate analysis (PCoA) plots colored by species. **(A)** Coordinate 1 is plotted along the y-axis, coordinate 2 is plotted along the x-axis. **(B)** Detail of clusters with negative PC1 and PC2 values. Pie charts indicate the numbers of each species belonging to designated clusters.

A principal component (PCoA) analysis was used to visualize the relationship between genotyped accessions (Figure 2; Supplementary File 3). In general species containing a D-genome (section Vertebrata) were distributed in discrete clusters along the PC2 axis. The diploid *Ae. tauschii* clusters are located furthest from other *Aegilops* species, the tetraploid D-genome species *Ae. crassa, Ae. cylindrica*, and *Ae. ventricosa* mid-way along the axis and the hexaploid *Ae. vavilovii* and *Ae. juvenalis* closest to other *Aegilops* species. *Aegilops tauschii* formed two discrete clusters reflecting the two separate gene pools sampled in the accessions (Dvorak et al., 1998b; Mizuno et al., 2010; Jones et al., 2013). Along the PC1 axis the bread wheat B-genome progenitor species *Ae. speltoides* clustered furthest from other *Aegilops* species and the other samples clustering with positive PC1 values belong to *Ae. mutica*. The remaining samples cluster more closely with negative PC1 and PC2 values. They are split into two locations; one consisting of diploid S, C, M, and N genome species; the other of diploid and tetraploid U genome containing species (section *Polyeides*).

A comparison of genetic differentiation and Fst between species (Table 2) revealed relationships between species with *Ae. tauschii* in particular showing a high degree of differentiation to most other species (except the related *Ae. crassa, Ae. ventricosa*, and *Ae. cylindrica*). Diploid species of different genome classifications showed a high degree of differentiation to each other but more similarity to polyploid species from the same section (e.g., *Ae. umbellulata* and polyploid species in section *Polyeides*) or containing a common genome (e.g., U-genome containing tetraploids). Other relationships are also revealed; the *Sitopsis* species showing greatest similarity to the S-genome of *Ae. kotschyi* and *Ae. peregrina* is *Ae. sharonensis*, while the UUMM tetraploid showing greatest similarity (Fst) to *Ae. juvenalis (*DDMMUU) is *Ae. geniculata* suggesting evolutionary relationships between these species.

**Table 2:** Genetic differentiation measured as Nei's standard genetic distance (above diagonal) and Fst measures (below diagonal) between species.

** 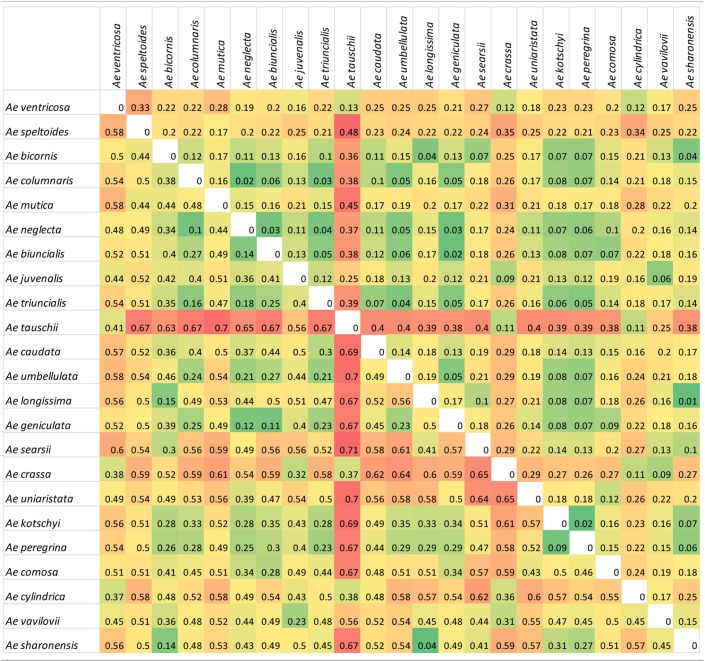 **

### Phylogenetic Relationships Within the Genus *Aegilops*

**Figure 3:**
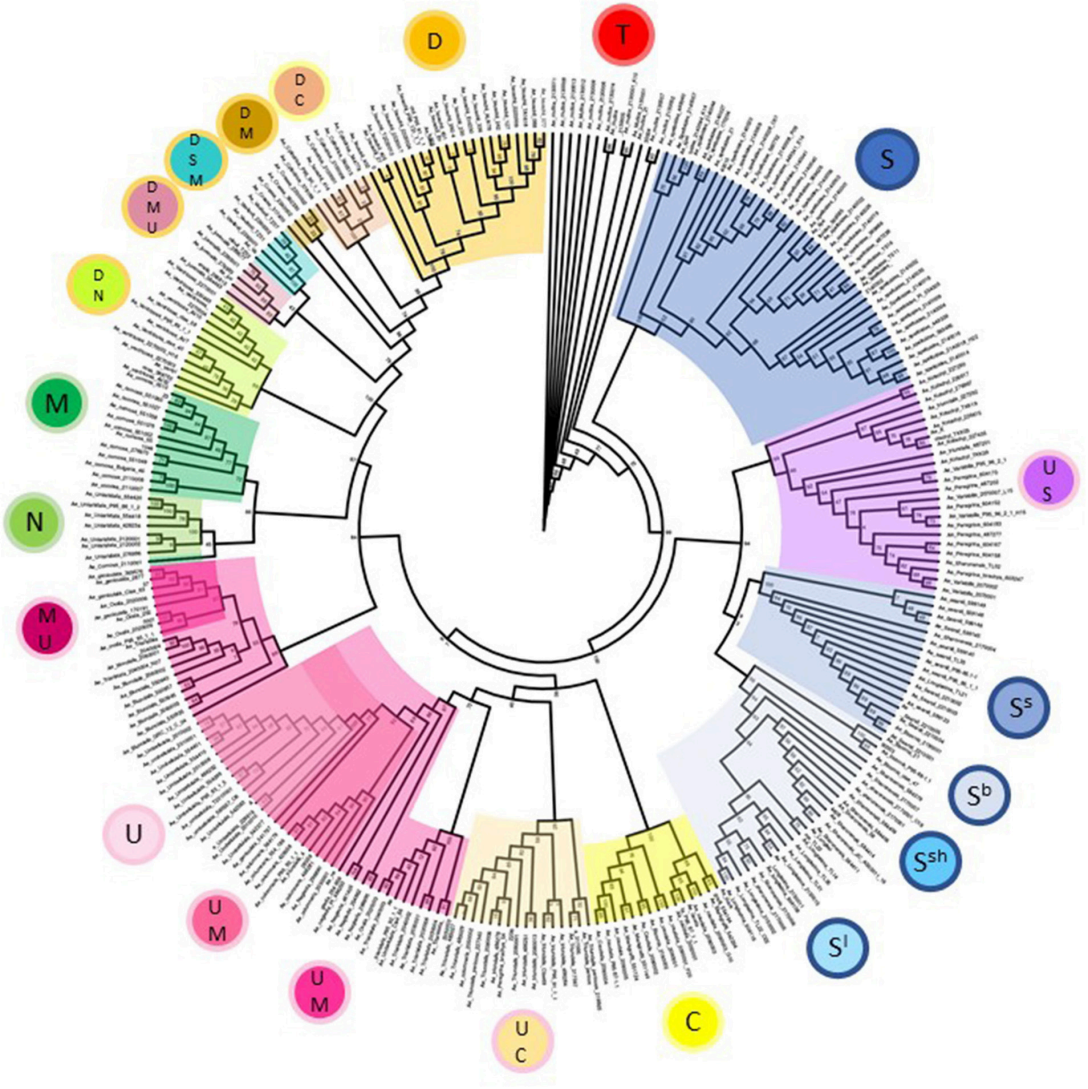
Phylogeny of *Aegilops* accessions used in the study based upon a maximum likelihood tree generated using all genotype data. Bootstrap support values are given at branch points and are based upon 10,000 replicates. Clades are colored according to genome designations given in the inner circles in Figure 1.

Phylogenetic analysis of *Aegilops* species used in the study broadly reflects the picture seen in the principal component analysis but allowed us to examine relationships between species and accessions in greater detail (Figure 3). All major branches had good bootstrap support with values over 80%. *Aegilops speltoides* accessions formed a separate clade to other S-genome species in the study at the base of the tree. Other S-genome containing species grouped together with tetraploids *Ae. kotschyi* and *Ae. peregrina* (UUSS) forming a separate clade to diploids *Ae. searsii, Ae. sharonensis, Ae. bicornis*, and *Ae. longissima*. The C-genome containing species *Ae. caudata* (diploid, CC) and *Ae. triuncialis* (tetraploid, UUCC) form two neighboring subclades of the clade also containing the U-genome containing diploid *Ae. umbellulata* (UU) and tetraploids *Ae. columnaris* and *Ae. neglecta* (tetraploid, UUMM). Interestingly, other UUMM tetraploids *Ae. geniculata, Ae. biuncialis*, and *Ae. ovata (syn. Ae. neglecta*, The Plant List) formed a separate clade to these, indicating diversity present in this group both within and between species. The diploid species *Ae. comosa* (MM) and *Ae. uniaristata* (NN) form neighboring subclades reflecting genetic similarity between these species. Finally, D-genome containing species were organized in a series of distinct subclades, including hexaploids *Ae. vavilovii* (DDSSMM) and *Ae. juvenalis* (DDMMUU); tetraploids *Ae. crassa* (DDMM), *Ae. cylindrica* (DDMM), and *Ae. ventricosa* (DDNN) and the diploid *Ae. tauschii* (DD). The *Ae. tauschii* subclade splits into the two distinct clusters also seen in the principal component analysis and relates to the geographic origin of the accessions selected (Dvorak et al., 1998b; Mizuno et al., 2010).

### Detecting *Aegilops* Introgressions in Wheat

**Figure 4:**
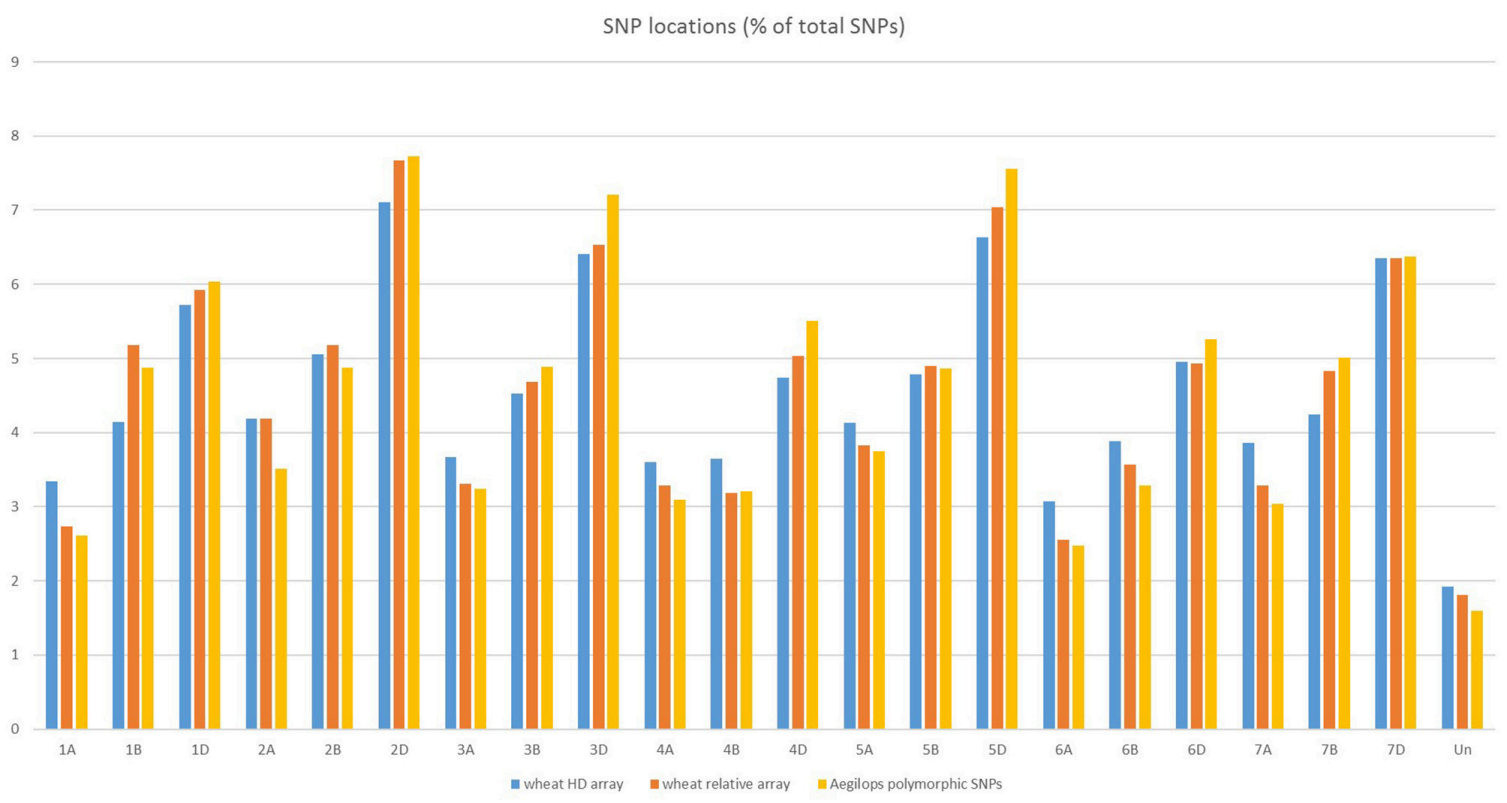
Chromosome locations of SNPs on the HD array, the wheat relative array and SNPs that are polymorphic between members of the genus *Aegilops* and *Triticum aestivum* from the wheat relative array.

**Figure 5:**
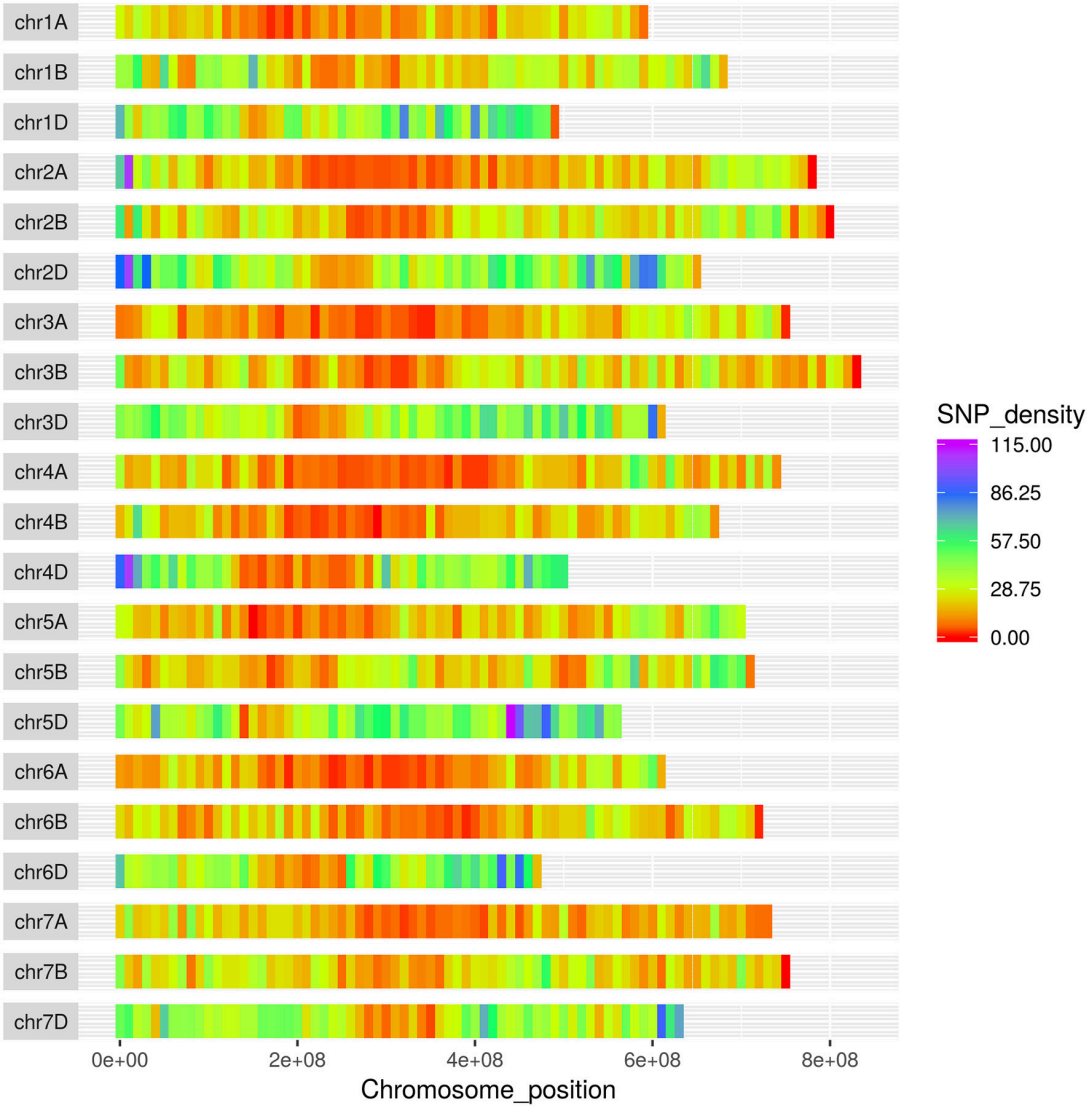
Distribution of SNPs from the Axiom® Wheat-Relative Genotyping Array in the wheat genome. The x-axis represents the physical distance along each chromosome, split into 10 Mbp windows.

The SNPs on the wheat relative array were assigned a putative chromosomal map location in wheat based upon BLAST alignment of the probe sequence surrounding each SNP against the Chinese Spring v 1.0 genome assembly (Winfield et al., 2016; The International Wheat Genome Sequencing Consortium (IWGSC), 2018). Over 98% of SNPs (36,009) on the wheat relative array were assigned a chromosome location (Supplementary File 4). The proportion of chromosome assignments were compared to the larger wheat HD array and calculated separately for SNPs that were polymorphic between the *Aegilops* and wheat on the wheat array (Figure 4). All three datasets had the highest proportion of SNPs located in the D-genome, then B genome, with the lowest proportions assigned to the A-genome. This was seen most dramatically in the set which were polymorphic between *Aegilops* and wheat (A genome 21.7%; B genome 31.0%; D genome 45.7%). The distribution of SNPs along chromosomes (Figure 5) demonstrated the higher proportion of SNPs in the D genome and also revealed a bias of SNP distribution toward the telomeres, as has been previously reported for exome based SNPs.

**Figure 6:**
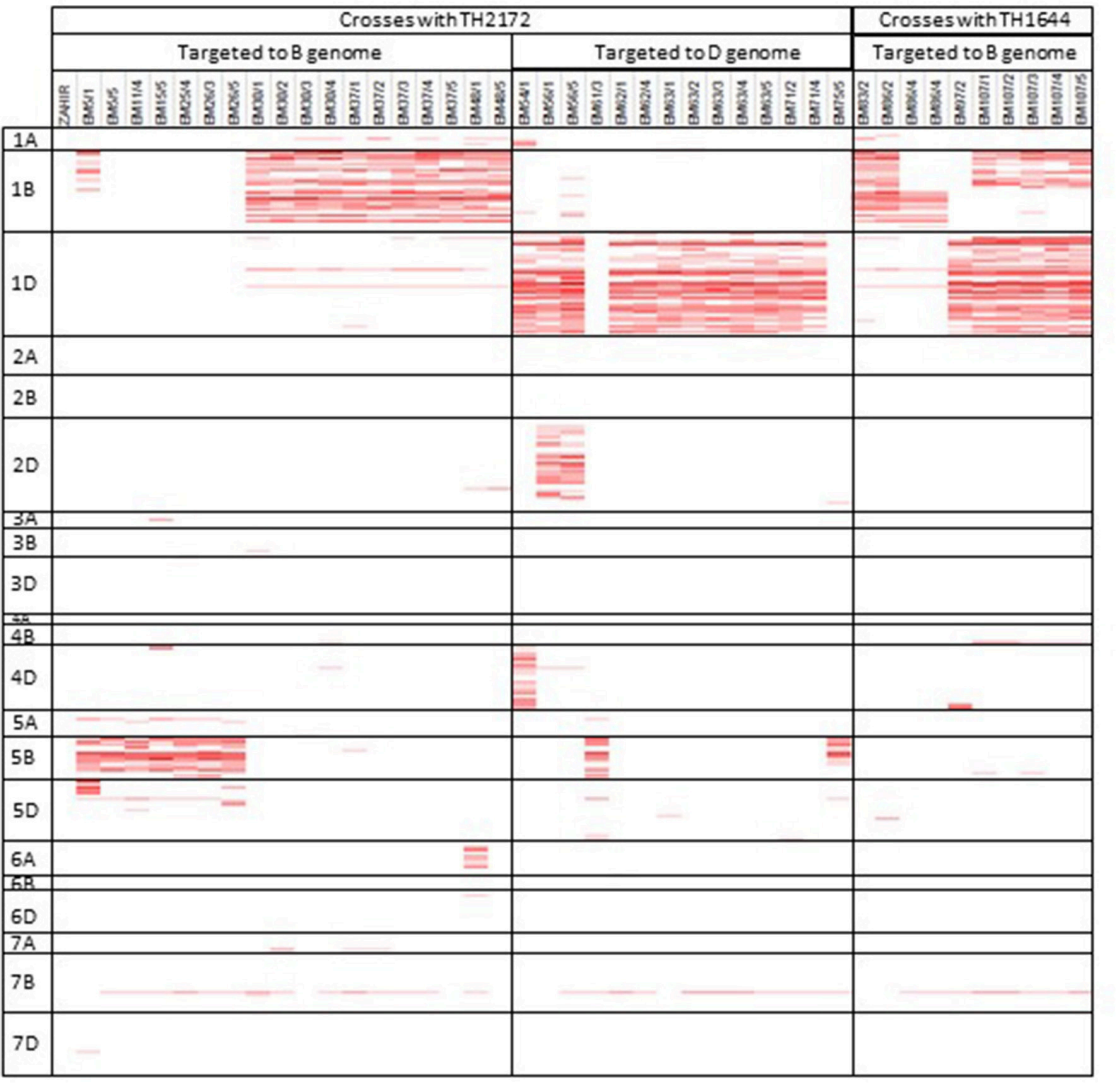
Identification of *Aegilops sharonensis* introgression segments in 41 recombinant wheat lines resistant to African *Puccinia graminis* f. sp. *Tritici*. Genotype calls from the array were compared to *Ae. sharonensis* over a 10 SNP window and a score of over 40% is considered indicative of introgressed material and is highlighted in red.

The SNP collection on the wheat relative array was employed in two different projects to identify introgressed segments of *Aegilops sp*. in a hexaploid wheat background. These projects took two different approaches to introducing the introgressions and in identifying the markers to track the introgressions. The first study used a specific accession of *Ae. sharonensis* to produce recombinant plants resistant to African Stem Rust. This project used the Axiom wheat HD array to identify and track introgressed regions by comparing the recombinant line and the *Aegilops sharonensis* genotype over 10 SNP windows (Millet et al., 2017). In this study we have shown that this analysis can be repeated using the subset of SNPs on the wheat relative array (Figure 6). The second project introgressed *Ae. speltoides* into a hexaploid wheat background with the aim of generating a population where individuals contained specific introgressed segments, which together represent the majority of the *Ae. speltoides* genome (King et al., 2017a). The approach taken to achieve this was to generate a genetic map containing 22,258 polymorphic SNPs (60.6% of total SNPs on the array) and refined this to 544 high quality framework markers. This map was used to inform and identify introgressed segments across individuals from five backcrossed populations, which were then confirmed by genomic *in situ* hybridization (GISH). A high frequency of introgressions were identified and it was possible to track these through the back-crossing process using markers from the wheat relative array.

## Discussion

This study describes the development and application of the first high-throughput genotyping platform specifically designed for screening wheat relative species. The Axiom® Wheat-Relative Genotyping Array contains a framework of over 36,000 SNP markers selected to be useful when screening a diverse range of species with a variety of genome structure and ploidy levels. The platform was used in this study to perform the largest screen of the genus *Aegilops* to date with multiple accessions representing all species in the genus. The array data was demonstrated to be useful for screening diversity within and between *Aegilops* species and was able to be used for examining relationships within the genus. Furthermore, the data was used to identify and track introgressions from *Aegilops* species in hexaploid wheat using two different approaches.

Over 94% of the SNPs on the array detected a polymorphism between *Aegilops* species. The average number of polymorphic SNPs within a species was 14,231 (36.8%) although the data suggested that when higher numbers of accessions were screened the number of polymorphic SNPs also increased. In the cases of *Ae. tauschii* (22) and *Ae. speltoides* (38) the number of polymorphisms detected doubled compared to random sets of five accessions. This suggests there is a high level of genetic diversity present in these species and careful selection of accessions will aid in sampling the variation present. The data revealed phylogenetic relationships between species in the genus *Aegilops*. As has been previously reported these data supported *Ae. speltoides* as the most basal species in the genus (Yamane and Kawahara, 2005). *Ae. speltoides* clustered separately to all other members of the Sitopsis section in both the PCA and phylogenetic analysis where it consistently was located at the base of the tree (99% bootstrap value).

The tetraploid U-genome containing species clustered closely on the PCA plot (Figure 2) and showed genetic similarity (Table 2). However, these species were clearly separated in the phylogenetic analysis where UUSS species *Ae. kotschyi* and *Ae. peregrina* are in a clade also containing S-genome diploids, whilst UUMM tetraploids located to the same clade as U-genome parent *Ae. umbellulata. Ae. triuncialis* (UUCC) located to a subclade between its two parental species *Ae. caudata* and *Ae. umbellulata*. Previous studies of the origin and evolution of polyploid *Aegilops* showed that the genomes of some species are very similar to those of the diploid progenitors, while other species are more modified (Kihara, 1954; Molnár et al., 2016). One theory of the process behind intraspecific divergence is that extinct species were the source of modified genomes or alternatively they were significantly rearranged during evolution. A third hypothesis is that the rate of parental genome modification in polyploids is different with one genome remaining similar to the parental genome (pivotal genome) and the second (differential genome) undergoing modification by complete or segmental chromosome substitutions (Zohary and Feldman, 1962; Kimber and Feldman, 1987; Badaeva et al., 2004). Two pivotal genomes (D and U) have been identified in *Aegilops* where genomes in all related species were similar to each other and to that of the parental diploid species, whereas the second genomes were modified compared to the original. Our data suggest the pivotal genome in polyploid UUMM species' *Ae. columnaris, Ae. biuncialis, Ae. neglecta* and *Ae. geniculata* is the U-genome, while *Ae. triuncialis* is found to be similar to both of its parental species, suggesting the C-genome has not diverged significantly as has been previously observed (Badaeva et al., 2004). However, *Ae. kotschyi* and *Ae. peregrina* cluster more closely with S-genome species suggesting that the U-genome in this instance is not pivotal.

Within the UUMM polyploid clade the four related tetraploid species are not organized into discrete sub-clades with and some mixing of species is seen within this clade and, where branching occurs, bootstrap support is not always high. This may suggest a polyphyletic origin of these species with different sources of parental genomes (one accession of *Ae. umbellulata* falls into a subclade of *Ae. neglecta*). Alternatively, this may indicate introgressive hybridization and gene flow between these genetically similar species. With multiple possible sources of the S-genome in *Ae. kotschyi* and *Ae. peregrina* the genetic diversity scores were compared and revealed the closest species to both were *Ae. sharonensis* and *Ae. longissima* as has previously been observed (Badaeva et al., 2004). When observed in the tree this result is explained by an *Ae. sharonensis* accession falling into the *Ae. peregrina* clade, a result which requires further investigation. Map locations of the SNPs on the Wheat-Relative array revealed a greater proportion were located on the D-genome than the A- or B-genomes. This contrasts with the usual results obtained when mapping polymorphisms in bread wheat, which are usually lacking in D-genome markers and reflects untapped diversity in D-genome relatives (Allen et al., 2016). The high level of intraspecific diversity present within *Ae. tauschii* and between this species and all others in the genus (except close relatives *Ae. crassa* and *Ae*. cylindrica) was observed in the PCoA analysis and high genetic diversity measures obtained (Figure 2; Table 2).

All *Aegilops* species screened had a high proportion of polymorphic SNPs when compared to bread wheat (average 88.8%) indicating a high level of potentially useful markers for detecting introgressions in a hexaploid wheat background. The specific number of polymorphic SNPs for a cross would depend upon the accession chosen and the wheat cultivar used. Although the array has been demonstrated to be useful in detecting diversity and relationships between a large collection of species, the real power and intended application of this tool is for specific intraspecific crosses where discrete populations are analyzed separately. In two specific case studies we have shown that the array may be applied to detecting and tracking *Aegilops* introgressions by two different strategies; in both cases multiple introgressions were detected. In the *Aegilops speltoides* project a high number of introgressions were detected and confirmed via GISH and additionally a gametocidal gene was detected (King et al., 2017a). In the *Ae. sharonensis* project recombinant chromosomes were identified and the gene conferring resistance to Ug99 group races was located on chromosome 1B, between 280 and 650 Mbp (Millet et al., 2017). The wheat-relative array has also been used in a third introgression project to screen wheat /*Am. muticum* (*syn. Ae. mutica*) recombinant chromosomes. The array enabled the identification and characterization of genome wide introgressions of various sizes (from large to very small) (King et al., 2017b).

The utility of the wheat relative genotyping array has been demonstrated to be effective in detecting both intra- and inter-specific diversity with insights into the structure and relationships within the genus *Aegilops*. The array data has been shown to be effective in identifying introgressed regions of *Aegilops* in a hexaploid bread wheat background. The application of the array extends beyond *Aegilops* and *Triticum*, having been designed for a wide range of species including those outside of these two genera. By linking with additional resources, such as the annotated genome sequence for wheat (The International Wheat Genome Sequencing Consortium (IWGSC), 2018) and with the development of technologies that enable targeted and precise introgression the potential gain from introducing selected beneficial genes from wild relatives of wheat is an exciting and increasingly feasible prospect.

## Author Contributions

AP-A analyzed the data and wrote the manuscript. AB, DS, and LM prepared samples. AB and DS performed genotyping. AB performed PCoA. PW performed phylogenetic analysis. IK, JK, KE, and GB developed the genotyping platform. All authors discussed the results and contributed to the final manuscript.

### Conflict of Interest Statement

The authors declare that the research was conducted in the absence of any commercial or financial relationships that could be construed as a potential conflict of interest.
